# High frequency of balance abnormalities in Turner syndrome

**DOI:** 10.1016/j.bjorl.2025.101713

**Published:** 2025-09-10

**Authors:** Vanessa Brito Campoy Rocha, Raquel Mezzalira, Guita Stoler, Gil Guerra-Junior, Sofia Helena Valente de Lemos-Marini, Andréa Trevas Maciel-Guerra

**Affiliations:** a Universidade Estadual de Campinas (Unicamp), Faculdade de Ciências Médicas (FCM), Programa de Pós-Graduação em Ciências Médicas, Campinas, SP, Brazil; b Universidade Estadual de Campinas (Unicamp), Faculdade de Ciências Médicas (FCM), Departamento de Otorrinolaringologia, Cirurgia de Cabeça e Pescoço, Campinas, SP, Brazil; c Universidade Estadual de Campinas (Unicamp), Faculdade de Ciências Médicas (FCM), Grupo Interdisciplinar de Estudos da Determinação e Diferenciação do Sexo, Campinas, SP, Brazil; d Universidade Estadual de Campinas (Unicamp), Faculdade de Ciências Médicas (FCM), Departamento de Pediatria, Campinas, SP, Brazil; e Universidade Estadual de Campinas (Unicamp), Faculdade de Ciências Médicas (FCM), Departamento de Genética Médica e Medicina Genômica, Campinas, SP, Brazil

**Keywords:** Turner syndrome, Postural balance, Vestibular system

## Abstract

**Objective:**

To perform a thorough evaluation of body balance in Turner Syndrome, adding evidence to the already scarce data in literature regarding labyrinth impairment in these patients.

**Methods:**

Adolescent and adult patients and a control group of healthy women were subject to otolaryngological, audiological, and vestibular evaluations. The latter included electronystagmography with caloric and rotational testing, cervical vestibular-evoked myogenic potential and static posturography with dynamic tests.

**Results:**

Twenty-seven patients aged 15–33 years (mean: 21.9-years) were evaluated. Karyotype was 45,X in 13/27 cases. Dizziness was reported in eight patients and progressive hearing loss in ten. Audiometry was abnormal in 13/27 cases, six of them sensorineural, six conductive, and one with a mixed pattern. A balance abnormality was found in 21/27 patients (78%). Nineteen patients had vestibular lesion, [12 peripheral (57%), four combined (19%), three central (11%)] and two had only somatosensorial deficit. Non-vestibular abnormalities were found in 6 (22%) patients, five with somatosensorial deficit and one with visual dependence. Balance abnormalities were neither associated with karyotype (45,X vs. other karyotypes) nor with hearing impairment. Control group comprised 20 healthy women aged 27–35 years (mean: 30.5 years); 4/20 (25%) had a balance abnormality, two peripheral and two central causes, a frequency significantly lower than that of TS patients (p < 0.001).

**Conclusion:**

Despite the low frequency of self-reported vestibular and auditory symptoms, this study reveals a high rate of inner ear involvement among women with TS, in which both auditory and vestibular pathways are frequently affected, often in a subclinical or compensated manner. These findings reinforce the importance of comprehensive screening protocols and indicate that appropriate interventions, including vestibular rehabilitation, should be considered as components of clinical follow-up in this population.

**Level of Evidence:**

4.

## Highlights

• Most patients with Turner syndrome aged 15–33 years have a balance abnormality.
• Balance abnormalities are more frequently peripheral or combined.
• Abnormalities were neither associated with karyotype nor with hearing impairment.
• The high rate of inner ear involvement is often subclinical or compensated.

## Introduction

Turner Syndrome (TS) is a chromosomal anomaly characterized by the presence of one normal X chromosome and partial or complete loss of the second sex chromosome.^1^, ^2^, ^3^ In addition to the classic 45,X karyotype, mosaicism and structural anomalies of the sex chromosomes can also be found. In mosaics, patients present the 45,X lineage associated with lineages with two or more intact sex chromosomes (46,XX, 46,XY, 47,XXX, and 47,XYY).^1^^,^^2^ Structural anomalies of X or Y include isochromosomes of the long arm [46,X,i(Xq) or 46,X,i(Yq)], ring chromosomes [46,X,r(X) or 46,X,r(Y)], and short-arm deletions [46,X,del(Xp)], among others, with or without mosaicism with the 45,X lineage.^2^

Clinically, TS manifests with short stature, primary hypogonadism, and various congenital anomalies, acquired disorders and dysmorphic signs, such as a triangular face, low hairline, low-set ears with posterior rotation, high-arched palate, micrognathia, retrognathism and webbed neck.^2^

Hearing disorders are also frequently found in TS.^4^, ^5^, ^6^ Most patients with hearing impairment suffer from middle ear pathologies, such as recurrent otitis media or serous otitis media in childhood (55%‒78% of cases).^3^, ^4^, ^5^ In this group, conductive hearing loss is expected and persists into adulthood in 20% of cases.^3^ Sensorineural hearing loss is also common, and this type of hearing loss has different presentations.^1^^,^^7^ The most common form is characterized by progressive involvement of high frequencies, similar to presbycusis.^6^^,^^7^ The second most common audiometric pattern is a notch in the mid frequencies.^7^ Sensorineural hearing loss may occur in up to 78% of women with TS between the ages of 16 and 34.^8^

Body balance has also been evaluated in TS, though there are only a few studies published on this subject. The oldest study analyzed audiological and vestibular findings in 93 adolescents and adult women undergoing hormone therapy, including 10 with TS.^9^ There was low labyrinth response in the caloric test in participants with TS, a finding not observed in the other patients.^9^ Another study applied a physical assessment to 184 girls with TS and observed that they had lower ability to maintain body balance compared to girls of the same age; however, no vestibular function assessments were conducted.^10^

A Swedish study revealed that women with TS performed worse in clinical body balance tests (Romberg test and Tandem Walk), and this deficiency was related to the severity of hearing loss.^11^ Another study analyzed balance in 19 women with TS using dynamic posturography and found worse results compared to controls, especially in conditions that assess vestibular function.^12^

A case report published in 2014 described a 46-year-old asymptomatic patient with a TS mosaic (45,X/46,XX), with bilateral vestibular hypofunction documented by the video head impulse test.^13^ The authors hypothesized that TS could be related to vestibulopathies due to estrogen deficiency.^13^

A North American self-report national survey conducted between 2016 and 2017 showed that perceived balance problems were significantly more common in TS compared to controls, especially in patients younger than 25-years-old.^14^ The odds of a prior fracture in those reporting balance problems was 54% greater than for those without balance problems.^14^

Balance dysfunction may have a negative impact in quality of life^15^ and bring a higher risk of falls with fractures in these patients, as bone demineralization is one of the complications of the syndrome.^12^^,^^14^ The aim of this study is to perform a thorough evaluation of body balance in TS, adding evidence to the already scarce data in literature regarding labyrinth impairment in these patients.

## Methods

This is a quantitative, descriptive, cross-sectional analytical study approved by local Research Ethics Committee (CAAE: 18344119.1.0000.5404). All participants over 18-years old and guardians provided informed consent by signing the Free and Informed Consent Form, and minors signed the Free and Informed Assent Form.

Our sample comprised adolescent and adult TS subjects who were initially evaluated at the outpatient clinic of the Hospital de Clínicas - UNICAMP and were under follow-up at the Endocrinology Unit, both from Hospital de Clínicas - UNICAMP, along with adult women included in the control group.

All TS patients had their diagnosis established by karyotype. Those with severe or profound hearing loss (pure-tone thresholds above 70 dB), uncontrolled comorbidities (diabetes, hypothyroidism, or migraine), severe bilateral visual impairment, active middle ear disease or other associated syndromes were excluded from this study.

The control group consisted of healthy women in the same age range, including medical residents and undergraduate students, who met the same exclusion criteria applied to TS patients. Control group was composed of healthy women with normal hearing and a normal Tympanometric curve (Type A).

### Initial evaluation

Age at evaluation, anthropometric data and karyotype were recorded. Participants underwent an otorhinolaryngological consultation, including history of hearing and balance symptoms, search for dysmorphic features and comprehensive audiometric evaluation (pure tone, speech and word recognition), audiometry and impedance, which are routine exams for monitoring TS patients.

### Balance assessment

All participants had their balance assessed using Electronystagmography (ENG) (Nistagmus Contronic®) with caloric and rotational testing, cervical Vestibular-Evoked Myogenic Potential (cVEMP) (MASBE Contronic®), and static Posturography (PG) with dynamic tests (Horus® platform).

Electronystagmography identifies labyrinthine lesions through oculomotor assessment (tracking, optokinetic nystagmus, saccades) and the presence of spontaneous or induced nystagmus (rotational and post-caloric). It evaluates the function of the lateral semicircular canals at low and medium stimulation frequencies (caloric and rotational tests, respectively).^16^

cVEMP records the response of sternocleidomastoid muscle ipsilateral to the stimulated ear. A high-intensity auditory stimulus is capable of exciting saccular nerve fibers, which send signals to the cervical musculature via the inferior vestibular nerve and vestibulospinal tract. This test assesses the integrity of saccular function at high frequencies.^16^

In turn, static posturography with dynamic testing evaluates the integration of proprioceptive, visual, and vestibular information. The participant stands on a platform while six conditions isolate each of the three systems responsible for body balance and verify the integrity of the vestibulospinal reflex. Ankle and hip strategies are tested to maintain balance on the platform.^16^

Test results were classified as “normal” or “abnormal”. In cases with abnormalities, the vestibular lesions were topographically classified as central, peripheral or combined. As the posturography also evaluates the three balance systems (vestibular, visual and proprioceptive), its non-vestibular abnormalities were described as somatosensory deficit and visual dependence.

The collected data are presented descriptively, followed by comparative analyses using the Chi-Square test or Fisher's exact test.

## Results

Our data are summarized in Table 1. A total of 27 TS patients aged between 15 and 33 years (mean: 21.9 years) were evaluated. Fifteen patients were between 15 and 21 years and 12 were more than 21 years old. The sample included 13 girls with a 45,X karyotype, six with structural abnormalities of the X chromosome, five with 45,X/46,XX mosaicism; and three with a normal or abnormal Y chromosome. The control group comprised 20 women aged between 27 and 35 years (mean: 30.5 years).

**Table 1:** Clinical data of the 27 patients with Turner syndrome and results of physical, audiological and vestibular evaluations.

Case	Age (years)	Karyotype	Dizziness	Hypoacusia	Weight (Kg)	Heigth (cm)	Dysmorphisms	Audiometry	ENG	cVEMP	Posturography	Lesion Site
1	15	45,X		Progressive asymmetric	52.4	144	Low-set ears Webbed neck	NL	R compensated vestibular hyporesponsiveness (LP 34% L)	NL	Abnormal - Conditions 1/2	P / SSD
2	15	45,X			48	143	Low-set ears Webbed neck	Mild CHL	NL	Reduced L	Abnormal - Condition 4	P
3	16	45,X	Recurrent spontaneous vertigo		45.9	140	Low-set ears Webbed neck High-arched palate	Moderate SNHL	L compensated hyperresponsiveness / R vestibular recruitment (LP 38% L and DP 30% L)	NL	NL	Combined – C / P
4	16	45,X	Recurrent spontaneous vertigo		46	148	Low-set ears Webbed neck High-arched palate	MILD SNHL	BL hyperresponsiveness	NL	NL	C
5	16	45,X / 46,X,+mar (Y-)			53.8	146	Low-set ears	NL	NL	NL	NL	No lesion
6	16	45,X / 46,XX			39.5	153	High-arched palate	NL	NL	NL	NL	No lesion
7	16	45,X / 46,XY / 47,XYY			37.9	133	Low-set ears	NL	L uncompensated vestibular hyperresponsiveness / L vestibular recruitment	NL	NL	Combined - C / P
8	17	45,X / 46,X, +mar (Y-)		Progressive symmetric	48	138	Webbed neck	NL	R compensated vestibular hyperresponsiveness / BL vestibular recruitment	Absent BL	NL	Combined – C /
9	18	45,X		Progressive asymmetric	51.9	148	Low-set ears Webbed neck	Mild CHL	NL	NL	Abnormal - Conditions 1/2	SSD
10	20	45,X			61.5	142		NL	NL	NL	NL	No lesion
11	20	45,X			64	148	Low-set ears Webbed neck	Mild CHL	NL	NL	NL	No lesion
12	20	45,X / 46,X,i(Xq)		Progressive symmetric	34.6	148		Mild CHL	NL	NL	NL	No lesion
13	21	45,X		Progressive symmetric	63	146	Low-set ears Webbed neck High-arched palate	Mild SNHL	NL	Absent BL	Abnormal - Limit of stability / Condition 1	P / SSD
14	21	45,X / 46,X,i(Yq)			78	151		NL	L compensated vestibular hyporesponsiveness (LP 43% R / DP 19% R)	NL	NL	P
15	21	45,X / 46,XX	Recurrent spontaneous vertigo		45.4	145	Low-set ears Webbed neck	NL	NL	Absent BL	NL	P
16	23	45,X / 46,X, +mar (Y+)			46.8	147	Low-set ears	NL	NL	NL	Abnormal - Condition 1/2	SSD
17	23	45,X / 46,XX	Recurrent spontaneous vertigo		54	156		NL	NL	Absent BL	NL	P
18	24	45,X / 46,X,i(Xq)	Recurrent spontaneous vertigo	Progressive symmetric	55.5	149		Moderate SNHL	R compensated vestibular hyperresponsiveness (LP 43% R)	NL	Abnormal - Limit of stability / Condition 1	C / SSD
19	25	45,X		Progressive symmetric	62	150	Low-set ears	Mild SNHL	R vestibular recruitment	Absent BL	NL	P
20	26	45,X			48	143	Low-set ears Webbed neck	NL	BL hyperresponsiveness	NL	NL	C
21	26	45,X / 46,X, r(X)	Recurrent spontaneous vertigo	Progressive asymmetric	47.9	135	Low-set ears High-arched palate	Mild SNHL	NL	NL	NL	No lesion
22	26	45,X / 46,XX	Recurrent spontaneous vertigo		63	148	Low-set ears	NL	NL	Absent BL	Abnormal - Conditions 3/6	P / VD
23	27	45,X			62.5	152	Webbed neck High-arched palate	Mild CHL	NL	Absent BL	NL	P
24	29	45,X			45	145	Low-set ears Webbed neck	NL	R compensated vestibular hyporesponsiveness (LP 45% L)	Reduced R	NL	P
25	30	45,X			47.3	146	Low-set ears Webbed neck	Mild CHL	Vestibular tone asymmetry (DP 26% R)	Reduced R	NL	Combined - C / P
26	32	45,X / 46,X, +mar(Y-)		Progressive symmetric	46.2	134	Low-set ears Webbed neck	Moderate MHL	Rotational test asymetry / L uncompensated vestibular hyporesponsiveness (LP/DP 26% R)	Absent L	Abnormal - All conditions	P
27	33	45,X / 46,XX	Chronic unsteadiness	Progressive symmetric	58.7	147	Low-set ears	NL	BL hyporesponsiveness	NL	NL	P

BL, Bilateral; C, Central; CHL, Conductive hearing loss; cVEMP, Cervical Vestibular-Evoked Myogenic Potential; DP, Directional preponderance; ENG, Electronystagmography; L, Left; LP, Labyrinthine Preponderance; MHL, Mixed Hearing Loss; NL, Normal; P, Peripheral; R, Right; SNHL, Sensorineural Hearing Loss; SSD, Somatosensory Deficit; VD, Visual Dependence.

Eight TS patients reported dizziness, and ten reported progressive hearing loss. The most frequent dizziness pattern was recurrent vertigo (spinning sensation) of spontaneous onset. Chronic unsteadiness was also reported. Seven of the ten women with hearing complaints described symmetrical hearing loss, while in the remainder it was asymmetric.

Average height and weight in TS patients were 145 cm and 52 kg, and typical dysmorphic features included low-set ears (19/27), high-arched palate (6/27), and webbed neck (14/27), the latter being more common in those with a 45,X karyotype (11/13) than in those with other karyotypes (3/14) (p = 0.001).

Audiometry was abnormal in nearly half of the cases (13/27), including six with sensorineural hearing loss, six with conductive hearing loss, and one with a mixed pattern. Abnormalities were seen in 9/13 girls with a 45,X and 4/14 with other karyotypes (p = 0.057). Hypoacusis was not reported by six of the 13 patients with abnormal audiometry, including one with moderate sensorineural hearing loss. On the other hand, three girls with hearing complaints had normal audiometry. The frequency of hearing impairment among the 15 younger girls (7/15) did not differ from that of the older ones (6/12) (p = 0.863).

ENG was abnormal in 13/27 cases. Though oculomotor function was normal in all women, and none had spontaneous nystagmus, abnormalities were found in rotational and caloric tests, with a predominance of peripheral findings (9/13). Peripheral findings included unilateral or bilateral hyporeflexia and post-caloric vestibular recruitment (where labyrinthine responses in the cold test are more intense than in the warm test). Central alterations, such as caloric hyperreflexia, were found in six patients. Some patients exhibited both peripheral and central findings, classified as combined involvement. In patients with normal results, the lateral canal, superior vestibular nerve and central vestibular system were presumed to function normally.

cVEMP was abnormal in 11/27 cases, showing bilaterally reduced or absent amplitude in seven cases and unilaterally in four. Reduced or absent P13‒N23 wave suggests lesions in the inferior vestibular nerve pathway, which conducts saccular stimuli to the sternocleidomastoid muscle. Posturography was abnormal in eight patients, with frequent Condition 2 (C2) impairment, indicating somatosensory system dysfunction.

Thus, 21/27 patients (78%) had a balance abnormality. Nineteen patients had vestibular lesion [12 peripheral (57%), four combined (19%), three central (11%)] and two had only somatosensorial deficit). Non-vestibular abnormalities were found in 6 (22%) patients, five with somatosensorial deficit and one with visual dependence. Balance abnormalities were neither associated with karyotype (11/13 45,X girls and 10/14 with other karyotypes) nor with hearing impairment (11/14 girls with normal hearing and 10/13 girls with hearing loss). Regarding the type of hearing loss, a balance abnormality was found in 5/6 girls in which it was sensorineural and 5/7 in which it was conductive or mixed.

Regarding control group, posturography was normal in all cases, but ENG was abnormal in four, two peripheral and two central findings. One of the patients with abnormal ENG (left compensated vestibular hypofunction) also had abnormal cVEMP (reduced response in the left ear), while in all the others cVEMP had a normal result. Thus, 4/20 women in the control group (25%) had a balance abnormality, two peripheral and two central, and this frequency is significantly lower than that of TS patients (p < 0.001).

## Discussion

In this study, only a minority of patients reported dizziness, a finding consistent with previous research.^3^ A hypothesis to explain the low prevalence of vertigo complaints is early-onset inner ear involvement, possibly beginning in infancy, or a mild and progressive dysfunction. This would allow sufficient time for central compensation mechanisms to adapt, rendering deficits less perceptible.

Hearing loss was also infrequently reported, although half of the participants presented audiometric deficits, as expected based on previous literature.^3^^,^^5^^,^^17^ The incidence of ear and hearing problems is 35-fold higher in TS compared to general population.^17^ Sensorineural hearing loss in these patients indicates inner ear (cochlear) involvement, whereas conductive loss reflects middle ear dysfunction (ossicular chain, tympanic membrane, Eustachian tube), which is common in individuals with recurrent otitis media or chronic otitis media.^18^ A mixed pattern suggests a combined middle and inner ear pathology.

Craniofacial anomalies, characteristic of TS, predispose to Eustachian tube dysfunction and consequent hypoventilation of the middle ear.^19^ Malformations of the stapes associated with TS have also been reported, contributing to both conductive and mixed hearing loss.^20^ One study linked this hearing loss pattern to low levels of Insulin-like Growth Factor-1 (IGF-1), suggesting that growth hormone replacement may aid in management.^4^

In this present cohort, sensorineural and conductive hearing loss were equally prevalent, with six patients in each group. Hultcrantz also found a similar distribution among patients older than 16 years.^21^ However, most studies in TS populations report a predominance of sensorineural loss (67% vs. 18%),^22^ possibly due to older participants in those samples. Sensorineural hearing loss in TS appears to progress more rapidly (0.5–2.2 dB/year) than in the general population (0.2–0.4 dB/year),^23^ particularly at higher frequencies.^18^

Despite evidence of progressive hearing loss, no significant difference in audiometric impairment was found between younger (<21 years) and older (>21 years) participants, possibly due to the limited sample size and narrow age range (15–35 years).

Hearing loss seemed to be more common in patients with the 45,X karyotype compared to those with other chromosomal anomalies (p = 0.057), consistent with previous findings.^17^ The severity of sensorineural hearing loss and auricular malformations increases proportionally with the number of 45,X cells.^4^^,^^24^ Patients with 45,X and 46,X,i(Xq) karyotypes (complete Xp monosomy) showed poorer audiometric outcomes than those with mosaicism or other structural abnormalities of the X chromosome.^5^^,^^17^

The pathophysiology of hearing loss in TS remains incompletely understood. Some hypotheses include delayed cranial base growth due to SHOX gene haploinsufficiency,^25^ reduced amount of cochlear hair cells at birth,^26^ and impaired intrauterine development of the otic capsule due to low IGF-1 levels.^27^ There is currently no conclusive evidence regarding the impact of estrogen replacement or growth hormone therapy on auditory preservation.^18^

The auditory assessment of patients with TS should be initiated at birth, repeated every 2–3 years in childhood and adolescence and every five years in adults.^17^ Regular hearing screening is already recommended by several authors,^1^^,^^2^ but there was no recommendation of balance evaluation until the publication of the new European clinical practice guideline for TS in 2024.^17^ Vestibular testing recommendation was made based on a very low quality of evidence due to the scarcity of scientific articles about balance performance in these patients.

A meta-analysis of non-syndromic individuals showed that balance impairment is more frequent among those with hearing loss compared to normal-hearing individuals.^28^ This may be explained by the anatomical and physiological proximity of the vestibular and cochlear systems within the inner ear.^28^ Hearing loss may lead to decline in labyrinthine function and postural instability,^29^ as well as impaired motor development.^30^ Although individuals with TS typically exhibit normal intelligence, motor difficulties and visuospatial deficits may interfere with daily activities, including driving.^3^

Despite the low rate of dizziness or hearing complaints in this sample, test results showed that most participants ‒ regardless of age or karyotype ‒ exhibited some form of inner ear dysfunction. The predominance of peripheral vestibular impairment is consistent with the frequent cochlear abnormalities described in this population.^1^^,^^3^^,^^5^

Posturography findings were notable for frequent abnormalities in condition 2 (eyes closed on a firm surface), suggesting somatosensory system deficits. Vestibular dysfunction combined with proprioceptive impairment may significantly affect balance, leaving the visual system as the only accurate spatial input.^31^

An English study found a prevalence of hearing loss of 84% and fracture history of 32% in 177 women with TS aged 19–60 years. Hearing loss was a positive predictor of fracture risk.^22^ Balance impairments, often associated with hearing loss, may contribute to increased fall risk, which is worrying given the high prevalence of bone demineralization in this syndrome.

Contrary to literature, this study did not find a statistically significant correlation between hearing loss and vestibular deficit. Even among normoacusic women, a high number of vestibular abnormalities was detected. Of the 14 patients with normal audiometry, only four had normal vestibular function. The high prevalence of peripheral vestibular dysfunction in this cohort raises questions about the underlying mechanisms, such as estrogen deficiency.^13^ The hypothesis of estrogen-related modulation of labyrinthine function is supported by studies showing that estrogens influence both central and peripheral vestibular pathways. Low estrogen may be involved in the microcirculatory disturbance of the inner ear,^32^ increasing the risk of vestibular diseases. Animal studies also found that estrogen deprivation can induce changes in central vestibular pathways, vestibular nucleus and impair vestibule-ocular reflex adaptation.^33^ In TS, hypoestrogenism may contribute to altered vestibular responses, either through direct effects on inner ear structures or via impaired central processing of balance information.

These findings underscore the importance of a multidisciplinary approach in the management of individuals with TS, integrating otolaryngological, audiological, and vestibular evaluations as part of routine care. Early identification of vestibular dysfunction, even in asymptomatic individuals, may help prevent future complications related to postural instability, falls, and reduced quality of life. In addition, counseling about occupational hazards in balance deficits should be also recommended.

Moreover, the implementation of tailored vestibular rehabilitation programs may promote functional improvement and enhance patients’ confidence and autonomy in daily activities. Given the subtle and often compensated nature of vestibular deficits in TS, clinicians should maintain a high index of suspicion, particularly when patients report vague symptoms such as imbalance, clumsiness, or visual dependence.

Considering the evidence of early-onset inner ear involvement, longitudinal monitoring is advisable to detect progressive deterioration. Serial audiometric and vestibular assessments, including posturography, could be useful in identifying subclinical changes. Additionally, future studies should explore the potential role of hormonal factors ‒ particularly estrogen and growth hormone ‒ in the preservation of vestibular function, as well as their impact on neural plasticity and sensory integration.

Another point of interest is the correlation between balance impairment and skeletal fragility in TS. As postural instability increases fall risk, and bone demineralization is a known feature of the syndrome,^2^^,^^17^ addressing vestibular dysfunction could also have implications for fracture prevention. This emphasizes the need for coordinated care strategies involving endocrinologists, otolaryngologists, physiotherapists, and primary care providers.

## Conclusion

In conclusion, despite the low frequency of self-reported vestibular and auditory symptoms, the objective findings in this study reveal a high rate of inner ear involvement among women with TS. These results reinforce the importance of comprehensive screening protocols and suggest that both auditory and vestibular pathways are frequently affected, often in a subclinical or compensated manner. Evaluation and appropriate interventions, including vestibular rehabilitation, should be considered as components of clinical follow-up in this population.

## ORCID ID

Sofia Helena Valente de Lemos-Marini: 0000-0002-8289-5703

## Declaration of competing interest

The authors declare no conflicts of interest.
